# Youth Suicide and Preceding Mental Health Diagnosis

**DOI:** 10.1001/jamanetworkopen.2024.23996

**Published:** 2024-07-30

**Authors:** Sofia Chaudhary, Jennifer A. Hoffmann, Christian D. Pulcini, Mark Zamani, Matt Hall, Kristyn N. Jeffries, Rachel Myers, Joel Fein, Bonnie T. Zima, Peter F. Ehrlich, Elizabeth R. Alpern, Stephen Hargarten, Karen M. Sheehan, Eric W. Fleegler, Monika K. Goyal

**Affiliations:** 1 Department of Pediatrics and Emergency Medicine, Children’s Healthcare of Atlanta, Emory University School of Medicine, Atlanta, Georgia; 2 Department of Pediatrics, Ann & Robert H. Lurie Children’s Hospital of Chicago, Northwestern University Feinberg School of Medicine, Chicago, Illinois; 3 Department of Emergency Medicine and Pediatrics, University of Vermont Medical Center and Children’s Hospital, University of Vermont Larner College of Medicine, Burlington, Vermont; 4 Children’s Hospital Association, Lenexa, Kansas; 5 Department of Pediatrics, Section of Hospital Medicine, University of Arkansas for Medical Sciences, Little Rock; 6 Department of Pediatrics, Children’s Hospital of Philadelphia, University of Pennsylvania, Philadelphia; 7 Semel Institute for Neuroscience and Human Behavior, University of California at Los Angeles; 8 Section of Pediatric Surgery, CS Mott Children’s Hospital, University of Michigan Ann Arbor; 9 Department of Emergency Medicine, Comprehensive Injury Center, Medical College of Wisconsin, Milwaukee; 10 Department of Pediatrics, Harvard Medical School, Boston, Massachusetts; 11 Department of Emergency Medicine, Harvard Medical School, Boston, Massachusetts; 12 Department of Emergency Medicine, Massachusetts General Hospital, Boston; 13 Department of Pediatrics, Children’s National Hospital, George Washington University, Washington, DC

## Abstract

**Importance:**

Suicide is a leading cause of death among US youths, and mental health disorders are a known factor associated with increased suicide risk. Knowledge about potential sociodemographic differences in documented mental health diagnoses may guide prevention efforts.

**Objective:**

To examine the association of documented mental health diagnosis with (1) sociodemographic and clinical characteristics, (2) precipitating circumstances, and (3) mechanism among youth suicide decedents.

**Design, Setting, and Participants:**

This retrospective, cross-sectional study of youth suicide decedents aged 10 to 24 years used data from the Centers for Disease Control and Prevention National Violent Death Reporting System from 2010 to 2021. Data analysis was conducted from January to November 2023.

**Exposures:**

Sociodemographic characteristics, clinical characteristics, precipitating circumstances, and suicide mechanism.

**Main Outcomes and Measures:**

The primary outcome was previously documented presence of a mental health diagnosis. Associations were evaluated by multivariable logistic regression.

**Results:**

Among 40 618 youth suicide decedents (23 602 aged 20 to 24 years [58.1%]; 32 167 male [79.2%]; 1190 American Indian or Alaska Native [2.9%]; 1680 Asian, Native Hawaiian, or Other Pacific Islander [4.2%]; 5118 Black [12.7%]; 5334 Hispanic [13.2%]; 35 034 non-Hispanic; 30 756 White [76.1%]), 16 426 (40.4%) had a documented mental health diagnosis and 19 027 (46.8%) died by firearms. The adjusted odds of having a mental health diagnosis were lower among youths who were American Indian or Alaska Native (adjusted odds ratio [aOR], 0.45; 95% CI, 0.39-0.51); Asian, Native Hawaiian, or Other Pacific Islander (aOR, 0.58; 95% CI, 0.52-0.64); and Black (aOR, 0.62; 95% CI, 0.58-0.66) compared with White youths; lower among Hispanic youths (aOR, 0.76; 95% CI, 0.72-0.82) compared with non-Hispanic youths; lower among youths aged 10 to 14 years (aOR, 0.70; 95% CI, 0.65-0.76) compared with youths aged 20 to 24 years; and higher for females (aOR, 1.64; 95% CI, 1.56-1.73) than males. A mental health diagnosis was documented for 6308 of 19 027 youths who died by firearms (33.2%); 1691 of 2743 youths who died by poisonings (61.6%); 7017 of 15 331 youths who died by hanging, strangulation, or suffocation (45.8%); and 1407 of 3181 youths who died by other mechanisms (44.2%). Compared with firearm suicides, the adjusted odds of having a documented mental health diagnosis were higher for suicides by poisoning (aOR, 1.70; 95% CI, 1.62-1.78); hanging, strangulation, and suffocation (aOR, 2.78; 95% CI, 2.55-3.03); and other mechanisms (aOR, 1.59; 95% CI, 1.47-1.72).

**Conclusions and Relevance:**

In this cross-sectional study, 3 of 5 youth suicide decedents did not have a documented preceding mental health diagnosis; the odds of having a mental health diagnosis were lower among racially and ethnically minoritized youths than White youths and among firearm suicides compared with other mechanisms. These findings underscore the need for equitable identification of mental health needs and universal lethal means counseling as strategies to prevent youth suicide.

## Key Points

**Question:**

What characteristics of youth suicide decedents and suicide circumstances are associated with having a documented mental health diagnosis?

**Findings:**

This cross-sectional study of 40 618 youth suicide decedents from the Centers for Disease Control and Prevention National Violent Death Reporting System found 24 192 decedents (59.6%) had no previously documented mental health diagnosis and 19 027 (46.8%) died by firearm suicide. The odds of having a documented mental health diagnosis were lower among racially and ethnically minoritized youths and among youths who used firearms.

**Meaning:**

These findings suggest that a critical need exists for comprehensive youth suicide prevention strategies, including early identification of mental health concerns, equitable access to mental health services, and universal lethal means counseling.

## Teaser

This cross-sectional study assesses sociodemographics, precipitating circumstances, and suicide mechanisms associated with documented mental health diagnosis among youths aged 10 to 24 years who died by suicide.

## Introduction

Suicide is the second leading cause of death for US youths aged 10 to 14 years and the third leading cause of death for youths aged 15 to 24 years, with nearly one-half due to firearms.^1^ From 2010 to 2021, 71 820 youths aged 10 to 24 years died by suicide with a near 50% increase in annual suicide rates over this period.^1^ Prior studies indicate that less than one-half of youths who die by suicide have a previously documented mental health (MH) problem or diagnosis.^2,3^ No studies utilizing recent data have examined whether documentation of prior MH diagnosis varies by sociodemographic and clinical characteristics.

As youth suicide rates have increased, disparities have widened. Among racial and ethnic groups, American Indian and Alaska Native youths have the highest rate of suicide overall (41.9 per 100 000 youths in 2020) while the rate of suicide has risen the fastest among Black youths (6.9 per 100 000 youths in 2010 and 12.9 per 100 000 youths in 2020—an 87% increase).^1,4^ Racially and ethnically minoritized youths experience inequities in access to MH services, resulting in disparities in outcomes.^5,6^ These disparities persisted and widened after the onset of the COVID-19 pandemic, which brought social isolation and stressors at home, decreased access to timely MH services, and increased access to firearms.^7,8,9,10,11^ During the first year of the pandemic, there were significantly more suicides than expected among male youths, children aged 5 to 12 years, youths aged 18 to 24 years, non-Hispanic American Indian and Alaska Native youths, and Black youths, as well as more firearm suicides than expected.^12^

Despite shifting patterns of MH service use and increased firearm accessibility, few studies have evaluated which population subgroups are most likely to have a known MH diagnosis prior to youth suicide. Early identification and documentation of an MH disorder, a known factor associated with increased risk of suicide, may facilitate timely targeted suicide prevention efforts and access to MH services.^13,14^ In the context of increased firearm access, identification of these intervenable characteristics is vitally important for suicide prevention. Therefore, our objective was to examine the association of sociodemographic characteristics, suicide mechanism, clinical characteristics, and precipitating circumstances with having a documented MH diagnosis among youth suicide decedents.

## Methods

### Study Design and Data Sources

This retrospective, cross-sectional study was determined exempt from human participants research and the requirement of informed consent by the institutional review board of Emory University and followed the Strengthening the Reporting of Observational Studies in Epidemiology (STROBE) reporting guideline.^15^ The study population included US youths aged 10 to 24 years that died by suicide between January 1, 2010, and December 31, 2021. We used mortality data from the National Violent Death Reporting System (NVDRS) Restricted Access Database. NVDRS is a state-based surveillance system that collects data on all violent deaths including suicide, homicide, legal intervention death, unintentional firearm death, and death of undetermined intent that might have been caused by violence.^16^ Data are collected from 3 required data sources: death certificates, coroner and medical examiner records, and law enforcement reports.^16^ We included data from all available states and territories that contribute data to NVDRS, which increased from 16 states in 2010 to 49 states, the District of Columbia, and Puerto Rico in 2021 (eTable in Supplement 1).^17^ At the time of the analysis, NVDRS data were available through 2021.

Suicide cases were determined based on *International Classification of Diseases, Tenth Revision* (*ICD-10*) cause of death codes (X60-X84, Y87.0, and U03) and/or based on evidence from source documents, with the final manner of death assigned by trained NVDRS abstractors.^16^ Suicide was assigned if death resulted from use of force against oneself and a collection of evidence indicated that use of force was intentional.^16,18^

### Study Measures

The primary outcome was the presence of a previously documented MH diagnosis among youth suicide decedents. To identify this outcome, we utilized the MH problem variable defined by NVDRS as (1) the decedent has a current MH diagnosis as categorized by *Diagnostic and Statistical Manual of Mental Disorders Fifth Edition* (*DSM-5*), not including from alcohol or other substance dependence, or (2) source documents (death certificate, coroner or medical examiner report, and police report) list the decedent as being treated for an MH problem, potentially from family member report or current prescription.^18^

Sociodemographic characteristics included race (American Indian or Alaskan Native; Asian, Native Hawaiian, or Other Pacific Islander; Black; White; multiple race category, and other [defined as any race not otherwise specified] or unspecified), ethnicity (Hispanic or non-Hispanic), age group (10-14 years, 15-19 years, and 20-24 years), and sex (female and male). Race and ethnicity were reported from combined raw data available from source documents (including death certificates, coroner and medical examiner records, and law enforcement reports) and verified by NVDRS investigator teams. Acknowledging race and ethnicity are social constructs, and racially and ethnically minoritized populations often have inequitable access to MH services, race and ethnicity were included as covariates in the analyses.^19,20^

Clinical characteristics included MH variables such as depressed mood, suicidality (suicide disclosure, history of nonsuicidal self-harm or self-injury, history of suicidal thoughts, or attempts), and substance misuse (alcohol and/or substance abuse). These MH characteristics were considered separate from a documented MH diagnosis. Decedents were categorized as having depressed mood if the they were perceived by themselves or others as depressed at the time of death; the definition does not require a clinical diagnosis of depression or that depression directly contributed to death.^18^

Precipitating circumstances were identified per coroner or medical examiner and law enforcement reports as having contributed to suicide death. Decedents could have multiple precipitating circumstances. We included precipitating circumstances regarding interpersonal problems, other life stressors (such as criminal, civil legal, school, or financial) and recent crises (defined as within 2 weeks prior to death).

Suicide mechanisms were defined as firearms; poisonings; hanging, strangulation, or suffocation; and other (which included motor vehicle, falls, and sharp or blunt instruments). Location of suicide was categorized as home, other, or unknown.

### Statistical Analysis

We conducted descriptive analysis with counts and frequencies of suicide deaths by sociodemographic and clinical characteristics, precipitating circumstances, location, and mechanism. We performed χ^2^ tests of proportions to assess differences in the proportion of suicide decedents with and without a documented MH diagnosis by sociodemographics, mechanism, and location. We used multivariable logistic regression to determine sociodemographic and clinical characteristics, precipitating factors, and mechanisms associated with the presence of a documented MH diagnosis. We constructed a model using the full cohort and then performed stratified analysis by age group. All models were adjusted for race, ethnicity, sex, and age group. Results were reported as adjusted odds ratios (aORs) and 95% CIs. All hypothesis testing was 2-sided, with statistical significance set at *P* < .05. All statistical analyses were performed with SAS version 9.4 (SAS Institute). Data analysis was conducted from January to November 2023.

## Results

### Characteristics of Study Sample

We identified 40 618 suicide decedents aged 10 to 24 years (23 602 aged 20 to 24 years [58.1%]; 32 167 male [79.2%]; 1190 American Indian or Alaska Native [2.9%]; 1680 Asian, Native Hawaiian, or Other Pacific Islander [4.2%]; 5118 Black [12.7%]; 30 756 White [76.1%]; 1017 multiple race category [2.5%]; 673 other or unspecified race [1.7%]; 184 unknown race [0.5%]); 5334 Hispanic [13.2%]; and 35 034 non-Hispanic [86.8%]). Most suicides occurred at home (25 174 [64.8%]) (Table 1). The most common precipitating circumstances were intimate partner problems (10249 [25.2%]) and family relationship problems (4462 [13.3%]) (Table 2).

**Table 1.** Sociodemographic Characteristics and Suicide Mechanisms by Documented Mental Health Diagnosis

Characteristic	Participants, No (%)		
	Total (N = 40 618)^a^	Documented mental health diagnosis (n = 16 426)^b^^,^^c^^,^^d^	No documented mental health diagnosis (n = 24 192)^b^^,^^c^
Race			
American Indian or Alaska Native	1190 (2.9)	333 (28.0)	857 (72.0)
Asian, Native Hawaiian, or Other Pacific Islander	1680 (4.2)	591 (35.2)	1089 (64.8)
Black	5118 (12.7)	1655 (32.3)	3463 (67.7)
Multiple race category	1017 (2.5)	429 (42.2)	588 (57.8)
White	30 756 (76.1)	13 153 (42.8)	17 603 (57.2)
Other or unspecified^e^	673 (1.7)	211 (31.4)	462 (68.6)
Unknown	184 (0.5)	54 (29.3)	130 (70.7)
Ethnicity			
Hispanic	5334 (13.2)	1959 (36.7)	3375 (63.3)
Non-Hispanic	35 034 (86.8)	14 376 (41.0)	20 658 (59.0)
Unknown	250 (0.6)	91 (36.4)	159 (63.6)
Age group, y			
10-14	3171 (7.8)	1150 (36.3)	2021 (63.7)
15-19	13 845 (34.1)	5702 (41.2)	8143 (58.8)
20-24	23 602 (58.1)	9574 (40.6)	14 028 (59.4)
Sex			
Male	32 167 (79.2)	11 994 (37.3)	20 173 (62.7)
Female	8446 (20.8)	4429 (52.4)	4017 (47.6)
Unknown	5 (<0.1)	3 (60.0)	2 (40.0)
Suicide method			
Firearms	19 027 (46.8)	6308 (33.2)	12 719 (66.8)
Poisoning	2743 (6.8)	1691 (61.6)	1052 (38.4)
Hanging, strangulation, or suffocation	15 331 (37.7)	7017 (45.8)	8314 (54.2)
Other	3181 (7.8)	1407 (44.2)	1774 (55.8)
Unknown	336 (0.8)	3 (0.9)	333 (99.1)
Location of injury			
Home	25 174 (64.8)	11 076 (44.0)	14 098 (56.0)
Other	13 665 (35.2)	5194 (38.0)	8471 (62.0)
Unknown	1779 (4.4)	156 (8.8)	1623 (91.2)

^a^ Percentages by column.

^b^ Percentages by row.

^c^ *P* < .001 for all comparisons.

^d^ As reported in National Violent Death Reporting System data from coroner, medical examiner, or law enforcement reports.

^e^ Other included any race not otherwise specified. National Violent Death Reporting System follows US Department of Health and Human Services and Office of Management and Budget standards for race and ethnicity categorization.^21^

**Table 2.** Clinical Characteristics and Precipitating Circumstances by Documented Mental Health Diagnosis

Clinical characteristics	Participants, No. (%)		
	Total (N = 40 618)	Documented mental health diagnosis (n = 16 426)^a^	No documented mental health diagnosis (n = 24 192)
Depressed mood	11 644 (28.7)	5879 (35.8)	5765 (23.8)
Suicidality or self-harm			
Suicide intent disclosure	8948 (22.0)	4518 (27.5)	4430 (18.3)
Left suicide note	11 521 (28.4)	5358 (32.6)	6163 (25.5)
History of nonsuicidal self-harm or self-injury^b^	499 (7.5)	362 (13.9)	137 (3.4)
History of suicidal thoughts^c^	10 554 (31.5)	6650 (48.2)	3904 (19.9)
History of suicide attempt	7634 (18.8)	5610 (34.2)	2024 (8.4)
Substance problem			
Alcohol abuse problem	3560 (8.8)	1908 (11.6)	1652 (6.8)
Alcohol use in hours preceding death^d^	3786 (14.6)	1614 (15.1)	2172 (15.0)
Substance abuse problem	6282 (15.5)	3371 (20.5)	2911 (12.0)
Substance caused death	2545 (6.3)	1611 (9.8)	934 (3.9)
Precipitating circumstances			
Interpersonal problem			
Family relationship problem^c^	4462 (13.3)	2126 (15.4)	2336 (11.9)
Other relationship problem	2040 (5.0)	882 (5.4)	1158 (4.8)
Intimate partner problem	10 249 (25.2)	4026 (24.5)	6223 (25.7)
Experienced interpersonal violence	238 (0.6)	111 (0.7)	127 (0.5)
Perpetrator of interpersonal violence	691 (1.7)	240 (1.5)	451 (1.9)
Abused as a child	969 (2.4)	687 (4.2)	282 (1.2)
Other life stressors			
Suicide of family member or friend	1159 (2.9)	587 (3.6)	572 (2.4)
Disaster^c^	363 (1.1)	203 (1.5)	160 (0.8)
Criminal problem	2758 (6.8)	1028 (6.3)	1730 (7.2)
Civil legal problem	553 (1.4)	225 (1.4)	328 (1.4)
School problem	3340 (8.2)	1553 (9.5)	1787 (7.4)
Financial problem	1287 (3.2)	580 (3.5)	707 (2.9)
Job problem	2308 (5.7)	1065 (6.5)	1243 (5.1)
Death of family member or friend	1557 (3.8)	735 (4.5)	822 (3.4)
Eviction or loss of home	639 (1.6)	283 (1.7)	356 (1.5)
Crises within 2 wk prior to death			
Any crises	10 933 (26.9)	4757 (29.0)	6176 (25.5)
Alcohol problem^c^	234 (0.7)	129 (0.9)	105 (0.5)
Substance abuse problem^e^	309 (0.9)	139 (1.0)	170 (0.9)
Mental health^e^	495 (1.5)	495 (3.6)	0
Family relationship problem^c^	1450 (4.3)	674 (4.9)	776 (3.9)
School problem^c^	733 (2.2)	306 (2.2)	427 (2.2)
Intimate partner problem^c^	3893 (11.6)	1619 (11.7)	2274 (11.6)
Financial problem^c^	152 (0.5)	69 (0.5)	83 (0.4)
Criminal legal problem^c^	963 (2.9)	338 (2.4)	625 (3.2)
Civil legal problem^c^	121 (0.4)	48 (0.3)	73 (0.4)
Job problem^e^	497 (1.5)	237 (1.7)	260 (1.3)

^a^ As reported in National Violent Death Reporting System data from coroner or medical examiner or law enforcement reports.

^b^ Data for this variable were collected starting November 1, 2020. During that time, the total number of decedents was 6659; 2605 had a documented mental health diagnosis and 4054 had no documented mental health diagnosis.

^c^ Data for these variables were collected starting August 1, 2013. During that time, the total number of decedents was 33 456; 13 805 had a documented mental health diagnosis and 19 651 had no documented mental health diagnosis.

^d^ Data for these variables were collected starting August 1, 2016. During that time, the total number of decedents was 25 994; 10 704 had a documented mental health diagnosis and 15 290 had no documented mental health diagnosis.

^e^ Data for these variables were collected starting July 1, 2013. During that time, the total number of decedents was 33 594; 13 858 had a documented mental health diagnosis and 19 736 had no documented mental health diagnosis.

### Characteristics of Youths With and Without a Documented MH Diagnosis

Among youth suicide decedents, 16 426 (40.4%) had a documented MH diagnosis and 24 192 (59.6%) had no documented diagnosis. Across individual groups, White youths had the highest rate of MH diagnosis (13 153 youths [42.8%]) and American Indian or Alaska Native youths had the lowest rate (333 youths [28.0%]); among Hispanic youths, 1959 (36.7%) had an MH diagnosis. Slightly more than one-half of female youths had an MH diagnosis (4429 youths [52.4%]), compared with 11 994 male youths (37.3%) (Table 1). Compared with those without an MH diagnosis, decedents with an MH diagnosis had higher rates of depressed mood (5879 youths [35.8%] vs 5765 youths [23.8%]), suicidal intent disclosure (4518 youths [27.5%] vs 4430 youths [18.3%]), and history of suicidal thoughts (6650 youths [48.2%] vs 3904 youths [19.9%]). Table 2 shows the association of preceding MH diagnosis with suicidality and self-harm, substance problems, precipitating circumstances, and crisis within the 2 weeks prior to death.

**Figure 1.**
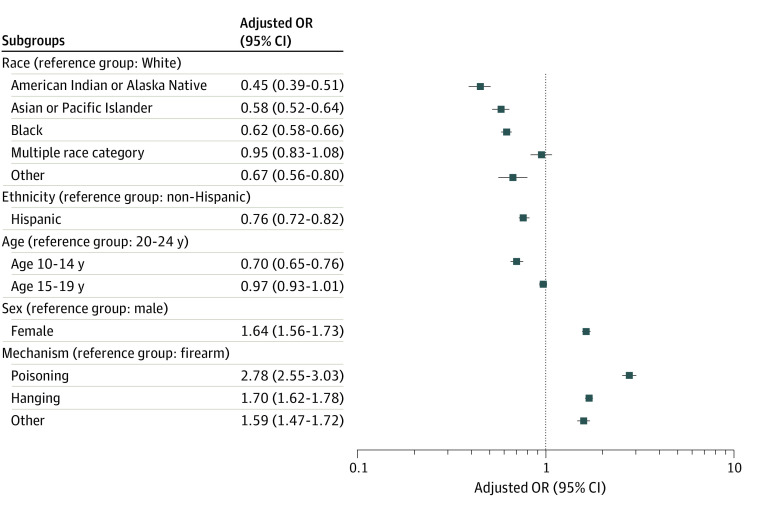
Multivariable Model for Sociodemographic Characteristics and Suicide Mechanisms Associated With Having a Documented Mental Health Diagnosis Logistic regression model with adjusted odds ratio adjusting for race, ethnicity, sex, and age. Pacific Islander included Native Hawaiian or Other Pacific Islander. Other race included any race not otherwise specified or unspecified race. Hanging included strangulation or suffocation. OR indicates odds ratio.

**Figure 2.**
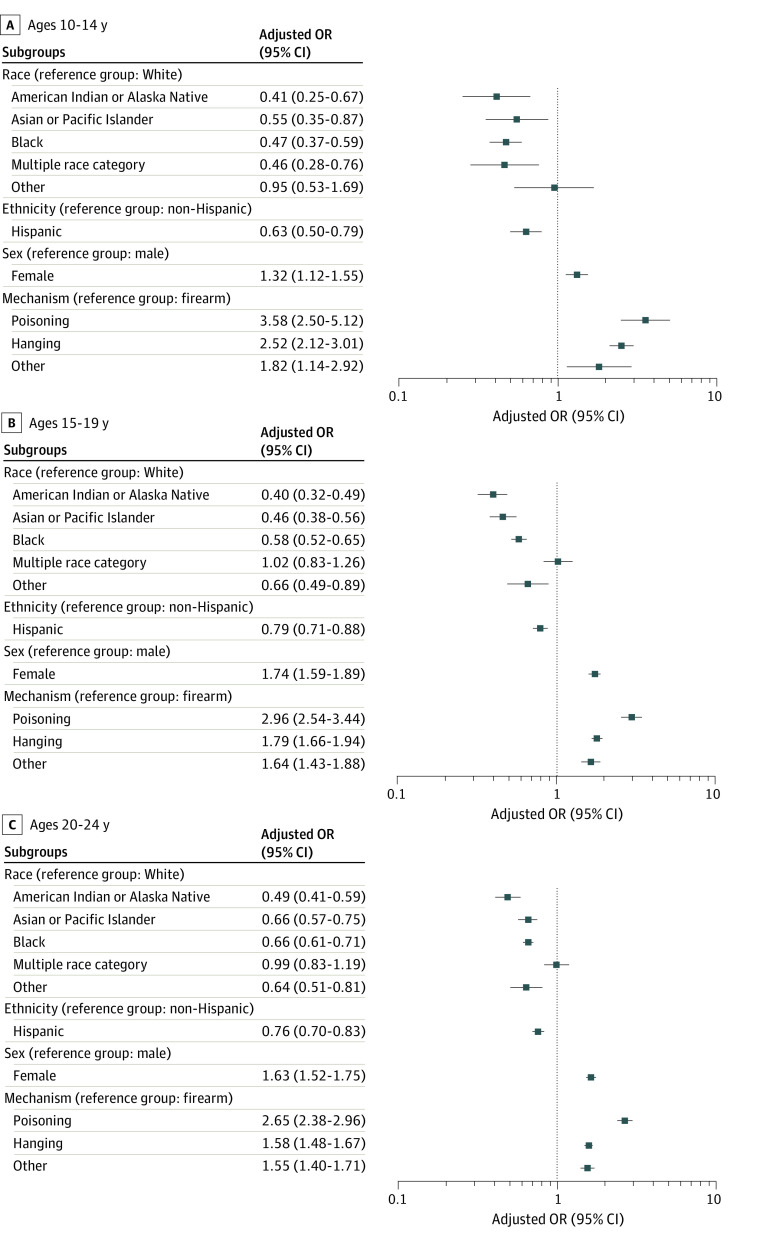
Multivariable Model for Sociodemographic Characteristics and Suicide Mechanism Associated With Having a Documented Mental Health Diagnosis Stratified by Age Logistic regression model with adjusted odds ratio adjusting for race, ethnicity, and sex. Pacific Islander included Native Hawaiian or Other Pacific Islander. Other race included any race not otherwise specified or unspecified race. Hanging included strangulation or suffocation. OR indicates odds ratio.

In comparison with White youths who died by suicide, there were lower adjusted odds of having a documented MH diagnosis among American Indian and Alaska Native youths (aOR, 0.45; 95% CI, 0.39-0.51); Asian, Native, Hawaiian, or Other Pacific Islander youths (aOR, 0.58; 95% CI, 0.52-0.64); and Black youths (aOR, 0.62; 95% CI, 0.58-0.66) (Figure 1). The ORs by race tended to narrow as youths aged, such as among Black youths aged 10 to 14 years (aOR, 0.47; 95% CI, 0.37-0.59), 15 to 19 years (aOR, 0.58; 95% CI, 0.52-0.65), and 20 to 24 years (aOR, 0.66; 95% CI, 0.61-0.71) (Figure 2). Compared with non-Hispanic youths, Hispanic youths had lower adjusted odds of having an MH diagnosis (aOR, 0.76; 95% CI, 0.72-0.82) (Figure 1). Compared with youths aged 20 to 24 years, the adjusted odds were lower for youths aged 10 to 14 years (aOR 0.70, 95% CI, 0.65-0.76) but were not statistically different for youths aged 15 to 19 years (aOR, 0.97; 95% CI, 0.93-1.01). Females had higher adjusted odds (aOR, 1.64; 95% CI, 1.56-1.73) of having a documented MH diagnosis compared with males .

There were higher adjusted odds of having a documented MH diagnosis among youths with depressed mood (aOR, 1.75; 95% CI, 1.67-1.83), with each suicidality or self-harm characteristic, and with substance abuse (aOR, 1.86; 95% CI, 1.76-1.97) when compared with youths without those characteristics (Table 3). Youths had higher odds of having a documented MH diagnosis if they had family relationship problems (aOR, 1.37; 95% CI, 1.29-1.47) or experienced child abuse (aOR, 3.39; 95% CI, 2.93-3.91) compared with youths without those precipitating circumstances. Youths with intimate partner problems had lower odds of having a documented MH diagnosis (aOR, 0.90, 95% CI, 0.86- 0.94) than youths without intimate partner violence problems.

**Table 3.** Multivariable Model for Clinical Characteristics and Precipitating Circumstances Associated With Having a Documented Mental Health Diagnosis

Clinical characteristics	Having a documented mental health diagnosis, aOR (95% CI)^a^^,^^b^	*P* value
Depressed mood	1.75 (1.67-1.83)	<.001
Suicidality or self-harm		
Suicide intent disclosure	1.68 (1.60-1.76)	<.001
Left suicide note	1.31 (1.25-1.37)	<.001
History of nonsuicidal self-harm or self-injury	3.96 (3.20-4.89)	<.001
History of suicidal thoughts	3.68 (3.51-3.87)	<.001
History of suicide attempt	5.37 (5.07-5.69)	<.001
Substance problem		
Alcohol	1.82 (1.70-1.96)	<.001
Alcohol use in hours preceding death	0.89 (0.83- 0.96)	.002
Substance abuse	1.86 (1.76-1.97)	<.001
Substance-caused death	2.28 (2.10-2.49)	<.001
Precipitating circumstances		
Interpersonal problem		
Family relationship problem	1.37 (1.29-1.47)	<.001
Other relationship problem	1.11 (1.01-1.21)	.03
Intimate partner problem	0.90 (0.86-0.94)	<.001
Experienced interpersonal violence	1.11 (0.85-1.44)	.45
Perpetrator of interpersonal violence	0.87 (0.74-1.02)	.09
Abused as a child	3.39 (2.93-3.91)	<.001
Other life stressors		
Suicide of family member or friend	1.47 (1.30-1.65)	<.001
Disaster	1.80 (1.45-2.23)	<.001
Criminal problem	0.93 (0.86-1.01)	.07
Civil legal problem	1.00 (0.84-1.19)	.99
School problem	1.40 (1.30-1.51)	<.001
Financial problem	1.20 (1.07-1.34)	.002
Job problem	1.29 (1.19-1.41)	<.001
Death of family member or friend	1.33 (1.20-1.47)	<.001
Eviction or loss of home	1.15 (0.98-1.35)	.01
Crisis within 2 wk prior to death		
Any crises	1.18 (1.13-1.24)	<.001
Alcohol problem	1.71 (1.32-2.22)	<.001
Substance abuse problem	1.13 (0.90-1.43)	.28
Mental health	NA	NA
Family relationship problem	1.29 (1.15-1.43)	<.001
School problem	1.06 (0.91-1.24)	.456
Intimate partner problem	0.98 (0.92-1.05)	.60
Financial problem	1.19 (0.86-1.64)	.30
Criminal legal problem	0.83 (0.72-0.95)	.006
Civil legal problem	0.92 (0.64-1.34)	.67
Job problem	1.33 (1.11-1.59)	.002

Abbreviation: NA, not applicable.

^a^ Adjusting for race, ethnicity, age, and sex.

^b^ As reported in National Violent Death Reporting System data from coroner or medical examiner or law enforcement reports.

### Mechanism

An MH diagnosis was documented for 6308 of 19 027 youths who died by firearms (33.2%); 1691 of 2743 youths who died by poisonings (61.6%); 7017 of 15 331 youths who died by hanging, strangulation, or suffocation (44.2%); and 1407 of 3181 youths who died by other mechanisms (44.8%). Among decedents with a documented MH diagnosis, the most common mechanism was hanging, strangulation, or suffocation (7017 decedents [42.7%]). Among decedents without a documented MH diagnosis, the most common mechanism was firearms (12 719 decedents [52.6%]). Among all decedents, 19 027 [46.8%] died by firearms. In the multivariable model, compared with youths who died by firearms, youths who died by poisonings (aOR, 2.78; 95% CI, 2.55-3.03); hanging, strangulation, or suffocation (aOR, 1.70; 95% CI, 1.62-1.78); and other mechanisms (aOR, 1.59; 95% CI, 1.47-1.72) had higher adjusted odds of having a documented MH diagnosis (Figure 1). Likewise, within each age group stratum, youths who died by poisonings and hanging, strangulation, or suffocation had higher adjusted odds of having a documented MH diagnosis compared with those who died by firearms.

## Discussion

In this cross-sectional study of youth suicides across the US that utilized the most recently available NVDRS data, we found that approximately 3 of 5 youth suicide decedents did not have a documented MH diagnosis. Racially and ethnically minoritized, male, and younger youths were less likely to have a documented MH diagnosis prior to suicide death than White, female, and older youths, respectively. Furthermore, youths who used a firearm, the mechanism with the highest case fatality rate,^22,23^ were far less likely to have a documented MH diagnosis.

Despite older literature that demonstrated that many youths who die by suicide have not received adequate MH screening and services, it appears that challenges persist in the identification of youths at risk even amid the current growing MH crisis. In this updated evaluation of suicide decedents within NVDRS, which captures the largest proportion to date (56.6% of US youth suicide decedents^1^), rates of documented MH diagnoses did not improve substantially from prior studies. From 2003 to 2012, only 34.6% of youth suicide decedents aged 5 to 11 years and 34.8% of youth suicide decedents aged 12 to 14 years had a documented known MH problem.^3,24^ Similarly, from 2013 to 2018, only 42.1% of youth suicide decedents aged 10 to 19 years had a known MH condition.^2^ This low rate of documented MH diagnosis among youth suicide decedents may reflect inadequate detection of MH needs, the impulsive nature of suicidal acts, increased access to more lethal means such as firearms, or alternative risk factors such as increased stressful life circumstances.^9,25,26^

We found significant racial and ethnic disparities in MH diagnoses among youths who died by suicide. This is consistent with prior literature^6^ that found only approximately 35% of Black youths had a documented current MH problem prior to death across age groups. Missed MH diagnosis among racially and ethnically minoritized youths who die by suicide may result from inequitable access to MH screening and diagnosis.^19^ On the other hand, racially and ethnically minoritized youths might have lower rates of MH diagnoses because they experienced other factors associated with increased risk of suicide besides MH conditions, including structural racism, discrimination, exposure to adverse childhood experiences, poverty, and lack of opportunity in certain neighborhoods.^27,28,29,30,31,32,33,34^ To counter these risk factors, suicide prevention efforts for racially and ethnically minoritized youths should include trauma-informed, culturally sensitive MH services, increased diversity in the MH workforce, and investments in school-based MH services, where Black youths are more likely than White youths to receive care.^19,35^ Such efforts could incorporate preventive interventions, grounded in cognitive-behavioral therapy, developed to counter stressors associated with systemic racism.^6,36^ Suicide prevention programming delivered in nontraditional community settings by individuals with lived experience may garner more receptivity and trust among Black youths than prevention efforts delivered in traditional health care settings.^32,37,38,39,40^

We found significant age differences in rates of MH diagnosis, with lower odds of having an MH diagnosis prior to suicide among youths aged 10 to 14 years compared with those aged 20 to 24 years. This finding is particularly notable because suicide rates have risen to become the second leading cause of death in youths aged 10 to 14 years.^1^ Among even younger children, during the first year of COVID-19 pandemic, non-Hispanic White 5- to 12-year-olds had a 31% increase in suicides over the expected number.^12^ Prior work suggests that suicide vulnerability may progress developmentally, from an impulsive response among younger children to a response to depressed mood or emotional distress during adolescence and adulthood.^3,24^ Suicide prevention strategies for young children in primary care and community settings should focus on fostering resilience, promoting peer and family connectedness, and empowering children with strategies to cope with stress and adversity.^14^

Regarding suicide mechanism, firearms were the most commonly utilized method among youths in our study, which differs from previous NVDRS work in which hanging, strangulation, and suffocation was the most common method among youths.^2,3^ This difference could be due to increased availability and accessibility of firearms over the course of our study period or inclusion of youths aged 20 to 24 years.^9,25^ Recent studies also demonstrate increased use of firearms as suicide mechanism.^12^ Similar to a prior study,^2^ we found that decedents without a documented MH diagnosis were far more likely to utilize a firearm than those with a documented MH diagnosis. Furthermore, in one study,^41^ 24% of teens and young adults spent less than 5 minutes between the decision to attempt suicide and the actual attempt. These impulsive attempts were more likely among those involved in a physical altercation and less likely among those who were depressed.^41^ This finding speaks to the need for universal lethal means counseling, delivered in community and school settings, regardless of whether youths have a known MH diagnosis. Teens should be involved in conversations about the risks of firearms given that many can easily access firearms.^42,43^ Among US teens, 44.2% perceived that they could access a firearm, while 20.2% perceived they could access a firearm within less than 5 minutes.^42^

Suicide prevention strategies are needed for the estimated 22.6 million US children living in households with firearms, of whom 4.5 million are exposed to firearms stored loaded and unlocked.^44^ More than 75% of guns used in youth suicide are owned by a family member, most commonly parents,^45^ and the presence of a firearm in the home is associated with an increased risk of youth suicide.^46,47,48^ This risk can be mitigated with secure firearm storage, including storing all guns locked, unloaded, with ammunition stored locked and in a separate location.^46^ Likewise, support and passage of state child access protection (CAP) laws, specifically negligence CAP laws, effectively reduce youth firearm suicide rates.^49,50^

Youths with depressed mood and suicidality characteristics, similar to prior literature,^2^ had higher odds of having a documented MH diagnosis. However, in our study, no MH diagnosis was documented in about one-half of youths noted to be depressed (at the time of or in the weeks leading up to death) or who had previously disclosed suicide intent within the month prior to death. Both nonsuicidal self-injury and previous suicide attempt are predictors of suicide among adolescents.^16,51,52,53^ However, at least 1 in 4 decedents with a history of nonsuicidal self-injury and 1 in 4 with a prior suicide attempt had no documented MH diagnosis. These youths presumably were not connected to MH services. Thus, attention is needed to increase accessibility of MH screening, diagnosis, and treatment in both primary care and specialty MH settings.

Family relationship and intimate partner problems were the most common circumstances experienced by youth decedents overall. Previous youth suicide studies have found higher rates of family and friend relationship problems among younger youths, while intimate partner problems were more prevalent in older age groups.^2,3,24^ Promoting connectedness and strengthening relationships among youths and parents or caregivers can protect against youth suicide.^24,54^ The Surgeon General 2021 call to action for suicide prevention^40^ delineates exemplars of evidence-based programs (eg, Sources of Strength and Good Behavior Game) that enhance social connectedness within families, school, and communities, including peer norm programs and community engagement activities.^55,56^

For precipitating circumstances among youth decedents in our study, the likelihood of having a documented MH diagnosis varied across family and life stressors, with no significant difference in the likelihood of having an MH diagnosis for any of the individual crisis variables. This speaks to youths’ challenges with adapting to family or life stressors and acute crisis regardless of the presence of an MH disorder. Additionally, teaching coping skills and increasing family and societal supports could prevent precipitating life circumstances.

### Limitations

There are limitations with this study. First, NVDRS is not a representative national sample because various states began contributing data during different years. However, NVDRS capture rates of youth suicide deaths in the Web-based Injury Statistics Query and Reporting System have increased from 28.0% in 2010 to 87.4% in 2021.^1^ Second, documentation of an MH diagnosis was defined by source records (ie, coroner, medical examiner, or law enforcement) and family member accounts; some decedents may have had an MH diagnosis that was unknown, undiagnosed, or unreported. However, utilization of these source records increases the likelihood of detecting MH diagnoses relative to administrative datasets that rely solely on billing diagnosis codes. Third, we could not conduct analyses using sexual orientation and gender identity variables due to a substantial degree of missingness. Fourth, the study was limited to analyses of quantitative data fields in NVDRS; incident narratives from law enforcement, coroner, and medical examiner reports were not examined for this study.

## Conclusions

In conclusion, most youth suicide decedents in this detailed, recent national sample did not have a documented MH diagnosis, signaling inadequate detection of MH needs. Youths who died by firearm suicide, the most common mechanism, had the lowest rate of documented MH diagnosis, highlighting the importance of universal lethal means counseling and CAP laws to increase barriers to firearm access. Social inequities may contribute to differences in MH diagnoses prior to suicide among racially and ethnically minoritized youths. These findings underscore the critical need to increase equitable access to MH screening, diagnosis, and treatment for all youths. Given the low rates of MH diagnoses among youth suicide decedents, prevention efforts must also address family and life stressors in tandem with MH risk factors. Both increased identification of unmet MH needs and universal, community-based approaches are needed to prevent youth suicide.

## Notes

Supplement 1. **eTable.** Availability of State, Territory, and Jurisdiction Data for NVDRS Web-based Injury Statistics Query and Reporting System (WISQARS) and Restricted Access Database (RAD)

Supplement 2.
Data Sharing Statement
