# Supplementary material for: Youth Suicide and Preceding Mental Health Diagnosis
Source: JAMA Netw Open. 2024 Jul 30;7(7):e2423996. doi: 10.1001/jamanetworkopen.2024.23996 (PMC11289695; doi:10.1001/jamanetworkopen.2024.23996)
Supplement: Supplement 1. — eTable. Availability of State, Territory, and Jurisdiction Data for NVDRS Web-based Injury Statistics Query and Reporting System (WISQARS) and Restricted Access Database (RAD) [file jamanetwopen-e2423996-s001.pdf]

Supplemental Online Content

Chaudhary S, Hoffman JA, Pulcini CD, et al. Youth suicide and preceding mental health diagnosis. *JAMA Netw. Open*. 2024;7(7):e2423996.
doi:10.1001/jamanetworkopen.2024.23996

eTable. Availability of State, Territory, and Jurisdiction Data for NVDRS Web-based Injury Statistics Query and Reporting System (WISQARS) and Restricted Access Database (RAD)

This supplemental material has been provided by the authors to give readers additional information about their work.

© 2024 Chaudhary S et al. *JAMA Netw Open.*

eTable. Availability of State, Territory, and Jurisdiction Data for NVDRS Web-based Injury Statistics Query and Reporting System (WISQARS) and Restricted Access Database (RAD)*

VDRS Program	2010	2011	2012	2013	2014	2015	2016	2017	2018	2019	2020	2021
**Alabama**									√	√	√	√
**Alaska**	√	√	√	√	√	√	√	√	√	√	√	√
**Arizona**						√	√	√	√	√	√	√
**Arkansas**											√	√
**California**								√[a]	√[a]	√[a]	√[a]	√[a]
**Colorado**	√	√	√	√	√	√	√	√	√	√	√	√
**Connecticut**						√	√	√	√	√	√	√
**Delaware**								√	√	√	√	√
**District of Columbia**								√	√	√	√	√
**Florida**											0[b]	0[b]
**Georgia**	√	√	√	√	√	√	√	√	√	√	√	√
**Hawaii**						√	√	0[b]	0[b]	√	0[b]	0[b]
**Idaho**											√	√
**Illinois**							√[c]	√[c]	√[c]	√[c]	√	√
**Indiana**							√	√	√	√	√	√
**Iowa**							√	√	√	√	√	√
**Kansas**						√	√	√	√	√	√	√
**Kentucky**	√	√	√	√	√	√	√	√	√	√	√	√
**Louisiana**									√	√	√	√
**Maine**						√	√	√	√	√	√	√
**Maryland**	√	√	√	√	√	√	√	√	√	√	√	√
**Massachusetts**	√	√	√	√	√	√	√	√	√	√	√	√
**Michigan**					√	√	√	√	√	√	√	√
**Minnesota**						√	√	√	√	√	√	√
**Mississippi**											√	√
**Missouri**									√	√	√	√
**Montana**										√	√	√
**Nebraska**									√	√	√	√
**Nevada**								√	√	√	√	√
**New Hampshire**						√	√	√	√	√	√	√
**New Jersey**	√	√	√	√	√	√	√	√	√	√	√	√
**New Mexico**	√	√	√	√	√	√	√	√	√	√	√	√
**New York**						√	√	√	√	0[b]	√	√
**North Carolina**	√	√	√	√	√	√	√	√	√	√	√	√
**North Dakota**										√	√	√
**Ohio**		√	√	√	√	√	√	√	√	√	√	√
**Oklahoma**	√	√	√	√	√	√	√	√	√	√	√	√

© 2024 Chaudhary S et al. *JAMA Netw Open.*

**Oregon**	√	√	√	√	√	√	√	√	√	√	√	√
**VDRS Program**	**2010**	**2011**	**2012**	**2013**	**2014**	**2015**	**2016**	**2017**	**2018**	**2019**	**2020**	**2021**
**Pennsylvania**							√[c]	√[c]	√[c]	√[c]	√	√
**Puerto Rico**								√	√	√	√	√
**Rhode Island**	√	√	√	√	√	√	√	√	√	√	√	√
**South Carolina**	√	√	√	√	√	√	√	√	√	√	√	√
**South Dakota**											√	√
**Tennessee**											√	√
**Texas**											√[a]	√[a]
**Utah**	√	√	√	√	√	√	√	√	√	√	√	√
**Vermont**						√	√	√	√	√	√	√
**Virginia**	√	√	√	√	√	√	√	√	√	√	√	√
**Washington**							√[c]	√[c]	√	√	√	√
**West Virginia**								√	√	√	√	√
**Wisconsin**	√	√	√	√	√	√	√	√	√	√	√	√
**Wyoming**										√	√	√
**TOTAL**	**16**	**17**	**17**	**17**	**18**	**27**	**32**	**37**	**41**	**44**	**50**	**50**

Abbreviations NVDRS: National Violent Death Reporting System; WISQARS: Web-based Injury Statistics Query and Reporting System; RAD: Restricted Access Database; VDRS: [State/Territory/Jurisdiction] Violent Death Reporting System

Key √: Data included. √ without a superscripted footnote denotes jurisdiction-wide data collection, which includes data in all counties within that jurisdiction.; 0: No data included

*Table adapted from Center for Disease Control and Prevention, National Violent Death Reporting System User Guidelines
[a] Some, but not all, counties in the state participated in data collection during this year.
[b] Excluded from this data year due to incomplete case reporting
[c] Collected data on >80% of violent deaths in the state, in accordance with requirements under which the state was funded.

© 2024 Chaudhary S et al. *JAMA Netw Open.*
