# Supplementary material for: Youth Suicide and Preceding Mental Health Diagnosis
Source: JAMA Netw Open. 2024 Jul 30;7(7):e2423996. doi: 10.1001/jamanetworkopen.2024.23996 (PMC11289695; doi:10.1001/jamanetworkopen.2024.23996)
Supplement: Supplement 2. — Data Sharing Statement [file jamanetwopen-e2423996-s002.pdf]

Data Sharing Statement

Chaudhary. Youth Suicide and Preceding Mental Health Diagnosis. *JAMA Netw Open*. Published July 30, 2024. doi:10.1001/jamanetworkopen.2024.23996

Data
Data available: No

Additional Information
Explanation for why data not available: Data is CDC National Violent Death Reporting System Restricted Access Data that cannot be shared for individual patient data
